# Simulation of the occipital transtentorial approach incorporating visualization of the cerebellar tentorium using three-dimensional computed tomography angiography and gadolinium-enhanced T1-weighted magnetic resonance imaging: technical note

**DOI:** 10.1007/s10143-023-02170-6

**Published:** 2023-09-29

**Authors:** Yuto Shingai, Masayuki Kanamori, Yoshiteru Shimoda, Shingo Kayano, Hitoshi Nemoto, Shunji Mugikura, Ryuta Saito, Teiji Tominaga

**Affiliations:** 1 Department of Neurosurgery, Tohoku University Graduate School of Medicine, Sendai, Japan; 2 Department of Radiology, Tohoku University Hospital, Sendai, Japan; 3 Department of Diagnostic Radiology, Tohoku University Graduate School of Medicine, Sendai, Japan; 4 Department of Neurosurgery, Nagoya University Graduate School of Medicine, Nagoya, Japan

**Keywords:** Occipital transtentorial approach, Preoperative simulation, Cerebellar tentorium, Bridging veins, Tentorial sinus

## Abstract

The occipital transtentorial approach (OTA) is one of the useful approaches to the lesions of the pineal region, dorsal brainstem, and supracerebellar region. However, a wide operative field is sometimes difficult to obtain due to the tentorial sinus and bridging veins. This study evaluated the usefulness of preoperative simulation of OTA, specifically including the cerebellar tentorium in 9 patients. All patients underwent computed tomography angiography and venography and gadolinium-enhanced three-dimensional T1-weighted magnetic resonance images (Gd-3D-T1WI). The images were fused, and the cerebellar tentorium, vessels, and tumor were manually extracted from Gd-3D-T1WI to obtain the simulation images. Visualization of the cerebellar tentorium could discriminate between bridging veins from the occipital lobe and cerebellum, and recognize the site of bridging to the tentorial sinus and variants which may interfere with the tentorial incision. Simulation of the tentorial incision was also possible based on the relationships between the tumor, tentorial sinus, bridging vein, and cerebellar tentorium. The simulation suggested that safe tentorial incision was difficult in two sides because of the crossed tentorial sinus draining the left basal vein and draining veins from the glioblastoma. The OTA was performed in eight cases, and no difficulty was experienced in the tentorial incision in all cases. The simulation findings of the bridging vein and tentorial sinus were consistent with the intraoperative findings. Preoperative simulation including the cerebellar tentorium is useful for determining the optimum and safe side and required extent of the tentorial incision necessary for tumor resection with the OTA.

**Supplementary Information:**

The online version contains supplementary material available at 10.1007/s10143-023-02170-6.

## Introduction

The occipital transtentorial approach (OTA) is commonly used to access the pineal region, splenium, dorsal brainstem, and supracerebellar region [1–8]. Appropriate incision of the cerebellar tentorium is essential to achieve safe and wide exposure of the lesion [9]. Therefore, determination of the optimum side and required extent of incision of the cerebellar tentorium requires preoperative understanding of the anatomical relationships between the cerebellar tentorium, tumor, and bridging veins from the occipital lobe or cerebellum and draining into the tentorial sinus.

Simulation based on three-dimensional (3D) computed tomography (CT) angiography (CTA), CT venography (CTV), and gadolinium-enhanced 3D T1-weighted magnetic resonance (MR) imaging (Gd-3D-T1WI) is considered to be a powerful tool for the preoperative evaluation of the anatomical structures in this region [2, 6, 10–12]. However, the relationships between the tumor, cerebellar tentorium, bridging veins from the cerebellar or occipital lobe to the tentorial sinus, and the presence of normal variants of vascular structures are sometimes difficult to understand because simulation of the cerebellar tentorium has never been investigated.

Here, we report our experience with simulation of the OTA using images of the cerebellar tentorium based on Gd-3D-T1WI with CTA and CTV, and discuss the usefulness for preoperative planning.

## Methods and materials

### Patients

Nine consecutive patients with tumors in the pineal region, supracerebellar region, or dorsal brainstem were treated in our department between 2019 and 2021.

### Simulation images

All patients underwent preoperative 3D plain CT, CTA, CTV, and Gd-3D-T1WI of the head. Subtraction processing of the CTA and CTV used a commercially available image processing workstation (Ziostation2, Ziosoft, Tokyo, Japan) to obtain the subtraction CTA and subtraction CTV. The plain CT, subtraction CTA, subtraction CTV, and Gd-3D-T1WI were then fused on the workstation. The cerebellar tentorium, vessels, and tumor were manually extracted from the Gd-3D-T1WI for fusion with the subtraction CTV to obtain the final simulation images. All CT scanning was performed with an ultra-high resolution CT scanner (Aquilion Precision CT, Canon Medical Systems Corporation, Otawara, Tochigi, Japan) and MR imaging with an MR system (Ingenia 3.0T CX, Philips Healthcare, Andover, MA).

### Determination of surgical approach and operative procedure

The approach route was determined based on the findings of the anatomical relationship between the tumor location and the distribution of the tentorial sinus and bridging veins from the occipital lobe and cerebellum.

The OTA was performed via unilateral occipital craniotomy. Before incision of cerebellar tentorium, the tentorial and straight sinuses and bridging vein from the occipital lobe were evaluated using the surgical microscope (OPMI PENTERO or KINEVO 900, Carl Zeiss Meditec Japan, Tokyo, Japan) and indocyanine green (ICG) video-angiography.

The suboccipital approach was performed via suboccipital craniotomy. During and after tumor resection, the bridging veins draining into the cerebellar tentorium were preserved to prevent damage to the drainers of the tumor and cerebellum.

### Validation of simulation images comparing intraoperative findings

Simulation images and intraoperative findings were reviewed to evaluate the usefulness of simulation images in planning tumor resection. First, the usefulness of the simulation of the cerebellar tentorium in determining the optimum route and required extent of incision of the cerebellar tentorium was assessed. Second, the agreement of the simulation images with the observed anatomical relationship between the tentorial sinus and bridging veins and the courses of tentorial and straight sinuses were examined. This study defined the tentorial sinus as coursing in the cerebellar tentorium and receiving flow from the cerebellum, occipital lobe, cerebral hemisphere, or brainstem, or without bridging vein [13]. We compared the presence or absence of tentorial sinus and the anatomical location between our preoperative simulation with the cerebellar tentorium and intraoperative findings from the operating microscope and ICG videoangiography. Similarly, we reviewed the presence or absence of bridging veins from the occipital lobe and cerebellum in the surgical fields and the site of bridging to the sinuses between the simulation and the intraoperative findings. We judged agreement between the simulation and intraoperative findings of the tentorial sinus and bridging vein as consistent, and the presence of structures only in the simulation or the intraoperative findings as inconsistent.

Anatomical relationships between the tentorial sinus and bridging veins were classified into four groups as previously reported [13]. Groups I, II, and III included the sinuses draining the occipital lobe, cerebellar hemisphere, and cerebellar tentorium, respectively. Group IV included tentorial sinuses originating from a vein bridging to the tentorial free edge.

## Results

### Patient characteristics

Patient characteristics are shown in Table 1. The six male and three female patients were aged from 7 to 71 years. Histological diagnosis was germ cell tumor in 5 cases, pilocytic astrocytoma in 2, glioblastoma in 1, and pineoblastoma in 1. Tumors were located in the pineal lesion in 6 cases, cerebellum in 2, and tectum in 1.

**Table 1:** Summary of the present patients

Case no.	Age/sex	Histological diagnosis	Tumor location	Side of tent	Findings of preoperative simulation					Approach side	Intraoperative findings						Approach to lesion
					Tentorial sinus	Normal variant	Tentorial sinus location to expected tentorial incision	Relationship between bridging vein and expected tentorial incision	Viability of approach		Microscopic				ICG video angiography		
											Tentorial sinus*	Bridging vein from occipital lobe*	Bridging vein from cerebellum	Straight sinus	Tentorial sinus*	Straight sinus	
1	9/M	Mixed germ cell tumor	Pineal	Right	Group II	No	Lateral and posterior	Separated	Possible	Yes	Consistent	Absent (consistent)	Not visible	Partially visible	Consistent	Partially visible	Possible
				Left	Group I	No	Lateral and posterior	Separated	Possible	No	N.E.						
2	34/F	Pilocytic astrocytoma	Tectum	Right	Group I	No	Lateral and posterior	Separated	Possible	Yes	Consistent	Present (consistent)	Absent	Completely visible	Consistent	Completely visible	Possible
				Left	Groups I and II	No	Lateral and posterior	Separated	Possible	No	N.E.						
3	29/M	Pineoblastoma	Pineal	Right	Group III	No	Lateral and posterior	No bridging vein	Possible	Yes	Consistent	Absent (consistent)	Absent	Completely visible	Consistent	Completely visible	Possible
				Left	Group III	No	Lateral and posterior	No bridging vein	Possible	No	N.E.						
4	11/M	Choriocarcinoma	Pineal	Right	Group II	No	Lateral and posterior	Separated	Possible	Yes	Consistent	Absent (consistent)	Not visible	Completely visible	N.E.	N.E.	Possible
				Left	Group IV	Yes	Crossing	Close**	Impossible	No	N.E.						
5	11/F	Pilocytic astrocytoma	Cerebellum	Right	Group II	No	Lateral and posterior	Separated	Possible	No	N.E.						
				Left	Group II	No	Lateral and posterior	Separated	Possible	Yes	Consistent	Absent (consistent)	Not visible	Completely visible	Consistent	Completely visible	Possible
6	7/M	Mature teratoma and germinoma	Pineal	Right	Group II	No	Posterior	Separated	Possible	No	N.E.						
				Left	Group II	No	Posterior	Separated	Possible	Yes	Consistent	Absent (consistent)	Not visible	Completely visible	Consistent	Completely visible	Possible
7	11/M	Immature teratoma	Pineal	Right	Group I	No	Lateral***	Separated	Possible	Yes	Consistent	Present (consistent)	Absent	Partially visible	No tentorial sinus	Partially visible	Possible
				Left	Group I	No	Lateral***	Separated	Possible	No	N.E.						
8	27/M	Immature teratoma	Pineal	Right	Group II	No	Posterior	Separated	Possible	No	N.E.						
				Left	Group II	No	Lateral***	Separated	Possible	Yes	Consistent	Absent (consistent)	Not visible	Completely visible	No tentorial sinus	Completely visible	Possible
9	71/F	Glioblastoma	Cerebellum	Right	Group II	No	Posterior	Not separated†	Impossible	No	Consistent^a^	Not visible^a^	Present^a^ (consistent)	N.E.	N.E.	N.E.	Possible^a^
				Left	Group II		N.A.			No							

*ICG* indocyanine green, *F* female, *M* male, *N.A.* not analyzed, *N.E*. not evaluated

*Whether consistent or inconsistent with the simulation with regard to the presence or absence of tentorial sinus or bridging veins from the occipital lobe and cerebellum, and the anatomical sites of these structures

**Basal vein drained directly into the tentorial sinus

***Tentorial sinus was located at a lateral site not exposed by the occipital transtentorial approach

^†^Draining vein of cerebellar glioblastoma was located just beneath the expected tentorial incision. ^a^Surgical view of suboccipital approach

### Determination of approach route based on simulation images

**Fig. 1:**
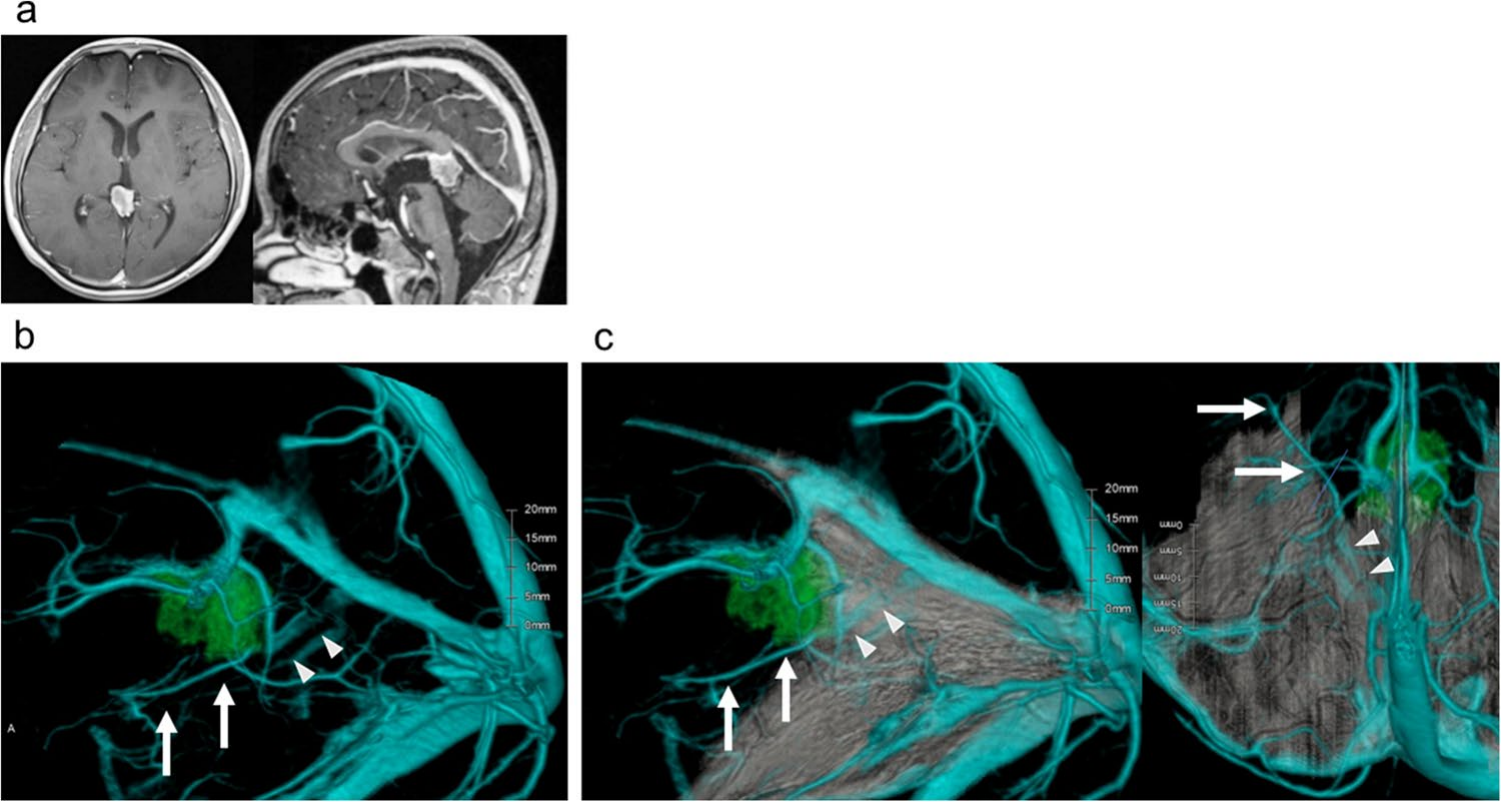
Case 4 with residual lesion after chemotherapy and radiation therapy for pineal choriocarcinoma. **a** Preoperative axial (left) and sagittal (right) gadolinium-enhanced T1-weighted MR images demonstrating the enhanced lesion in the pineal region. **b, c** Preoperative simulation of the left lateral views based on three-dimensional reconstruction with computed tomography venography and gadolinium-enhanced T1-weighted MR images demonstrating the anatomical relationships of the tumor (green), veins, and sinuses (blue) without (**b**) and with (**c**) the cerebellar tentorium. Left basal vein (arrows) directly drains into the tentorial sinus (arrowheads). The difficulty of tentorial incision for the occipital transtentorial approach on the left side was easy to recognize from the simulation with the cerebellar tentorium

**Fig. 2:**
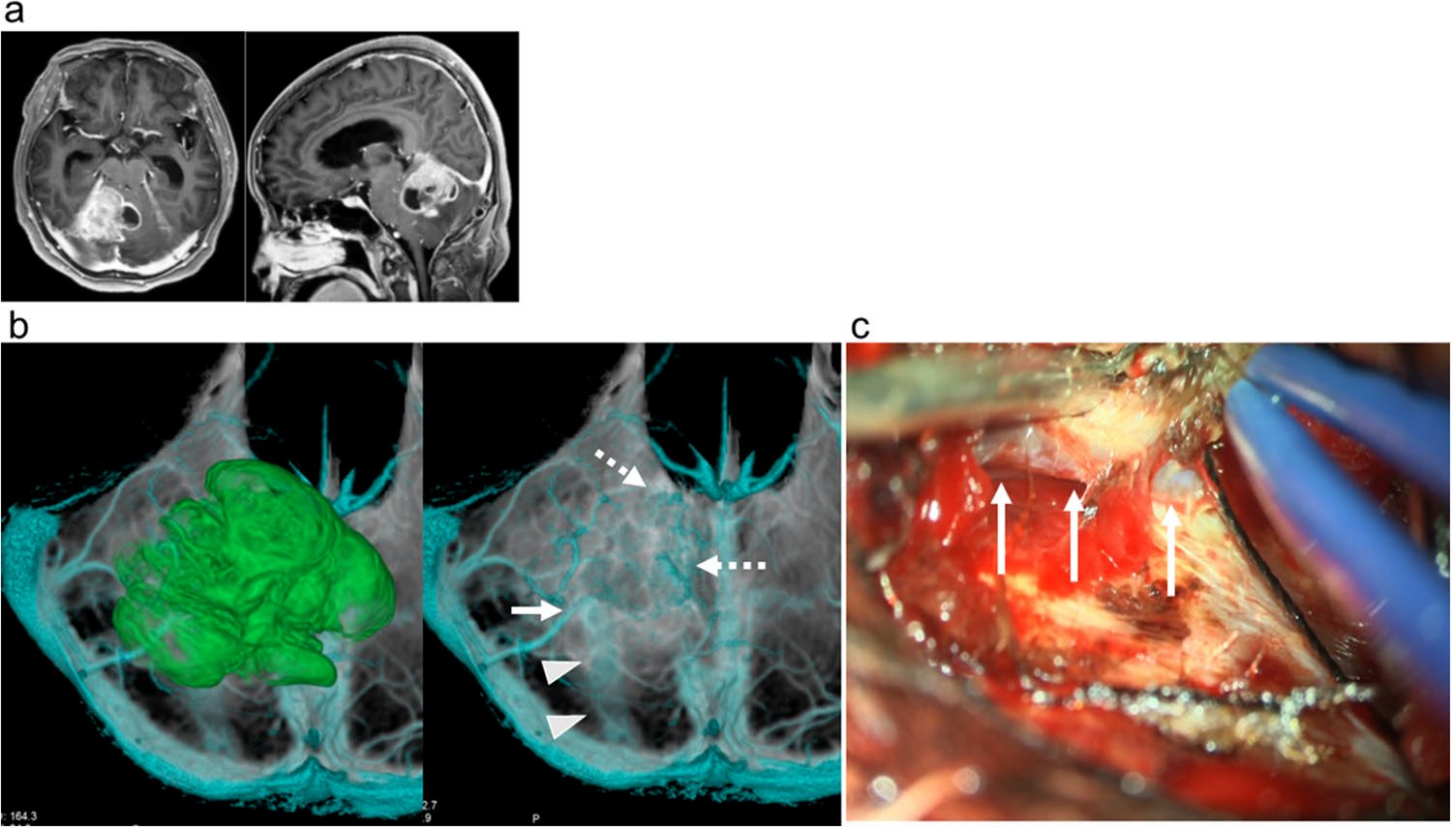
Case 9 with cerebellar glioblastoma. **a** Preoperative axial (left) and sagittal (right) gadolinium-enhanced T1-weighted MR images demonstrating the enhanced lesion in the right cerebellar hemisphere and cerebellar vermis. **b** Preoperative simulation of inferior views with (left) and without (right) the tumor based on three-dimensional reconstruction with computed tomography venography and gadolinium-enhanced T1-weighted MR images demonstrating the anatomical relationships of the veins and sinuses (blue), cerebellar tentorium (gray), and tumor (green). Tentorial sinuses (arrowheads) and bridging veins from cerebellum (arrow) are noted on the inferior views (arrows on upper middle). A number of draining veins from the glioblastoma were found anterior to the tumor (dashed arrows). These findings suggested that tentorial incision and retraction of the cerebellar tentorium at the OTA could damage the draining veins of the glioblastoma and cerebellum, and the patient underwent right suboccipital craniotomy and tumor resection. **c** Intraoperative microscopic view demonstrating the bridging vein from the cerebellum (arrow) medially draining into the tentorial sinus (arrows). This finding was consistent with the simulation images (arrow in **b**)

The simulation images including the cerebellar tentorium clearly demonstrated the tentorial sinus and the bridging veins draining into the tentorial sinus, compared to the simulation images without the cerebellar tentorium (Fig. 1 and Supplementary Figs. 1, 2, and 3). The preoperative findings of the tentorial sinus and its bridging veins and the presence of normal variants are summarized in Table 1. Based on these findings, the approach route and the side and range of tentorial incision were determined for the OTA. Findings that indicated the risk of postoperative complications were found in two of nine cases. A case of pineal choriocarcinoma indicated that tentorial incision might lead to injury of the basal vein or the tentorial sinus (Fig. 1). This rare normal variant was found in only one (5.5%) of 18 sides. In this case, the risk of tentorial incision was difficult to recognize based only on the simulation with venography without the cerebellar tentorium (Fig. 1b), but addition of cerebellar tentorium clarified the anatomical relationship of the planned tentorial incision, tumor, and basal vein and its draining sinus (Fig. 1c). A case of cerebellar glioblastoma suggested that tentorial incision and retraction of the cerebellar tentorium might damage the draining veins of the glioblastoma (dashed arrow in Fig. 2) at the early stage of surgery and those of the cerebellum draining into the tentorial sinus (arrow and arrowhead in Fig. 2). Therefore, right suboccipital craniotomy and tumor resection were selected instead of the OTA. Tumor resection were performed through the OTA in eight patients and the suboccipital approach in one.

**Fig. 3:**
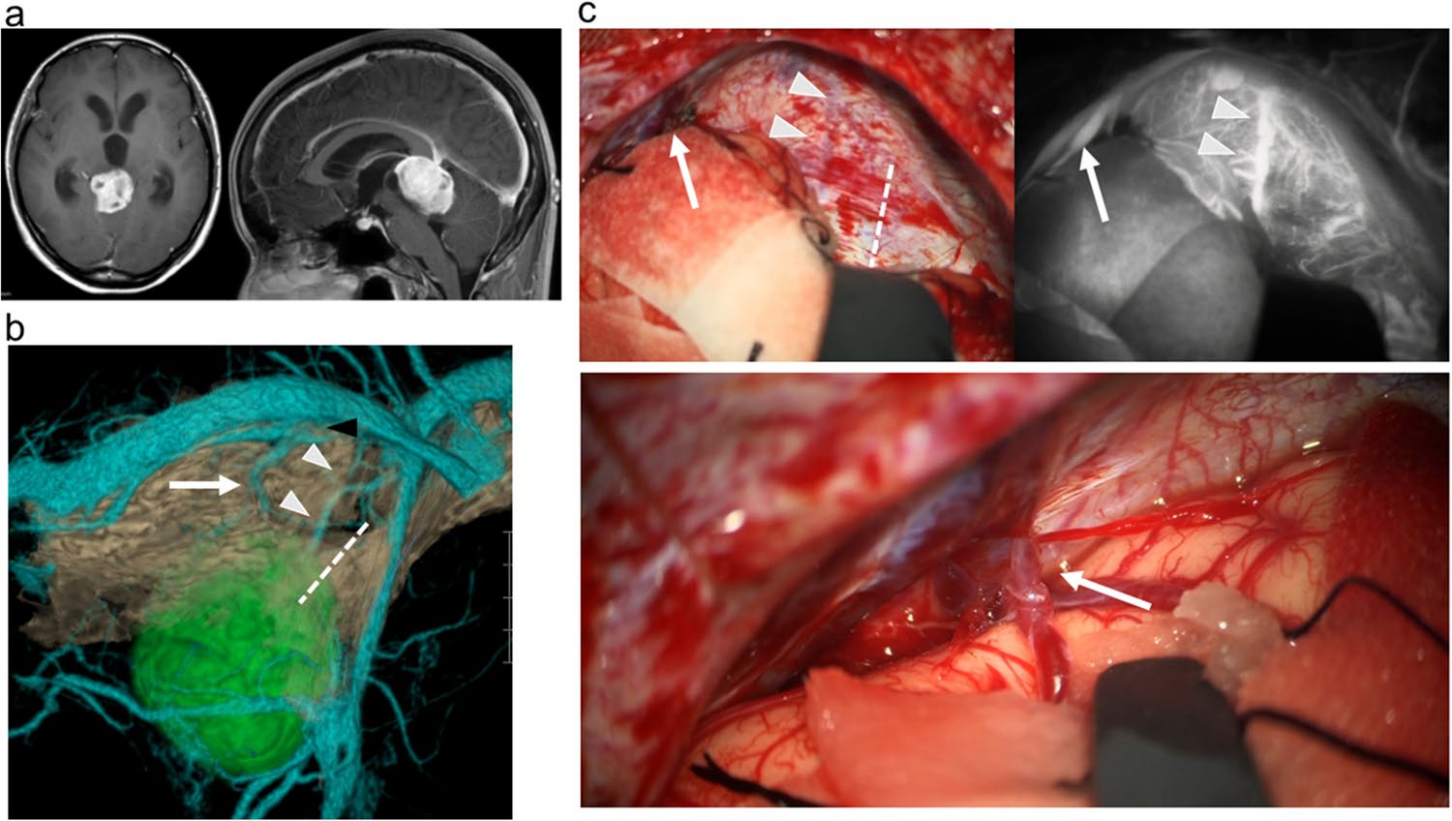
Case 2 with tectal pilocytic astrocytoma. **a** Preoperative axial (left) and sagittal (right) gadolinium-enhanced T1-weighted MR images demonstrating the enhanced lesion in the tectum of the midbrain. **b** Preoperative simulation of the posterolateral view based on three-dimensional reconstruction with computed tomography venography and gadolinium-enhanced T1-weighted MR images demonstrating the anatomical relationships of the tumor (green), veins and sinuses (blue), and cerebellar tentorium (brown). The small tentorial sinus (arrowhead) and the bridging vein from the occipital lobe (arrow) drained into the transverse sinus. The dashed line indicates the expected tentorial incision. **c** Intraoperative microscopic view (upper left and lower) and finding of indocyanine green video-angiography (upper right) demonstrating that the small tentorial sinus (arrowheads) and bridging vein from the occipital lobe (arrow) drained into the tentorial sinus consistent with the preoperative simulation. The dashed line in the upper left panel indicates the actual tentorial incision

**Fig. 4:**
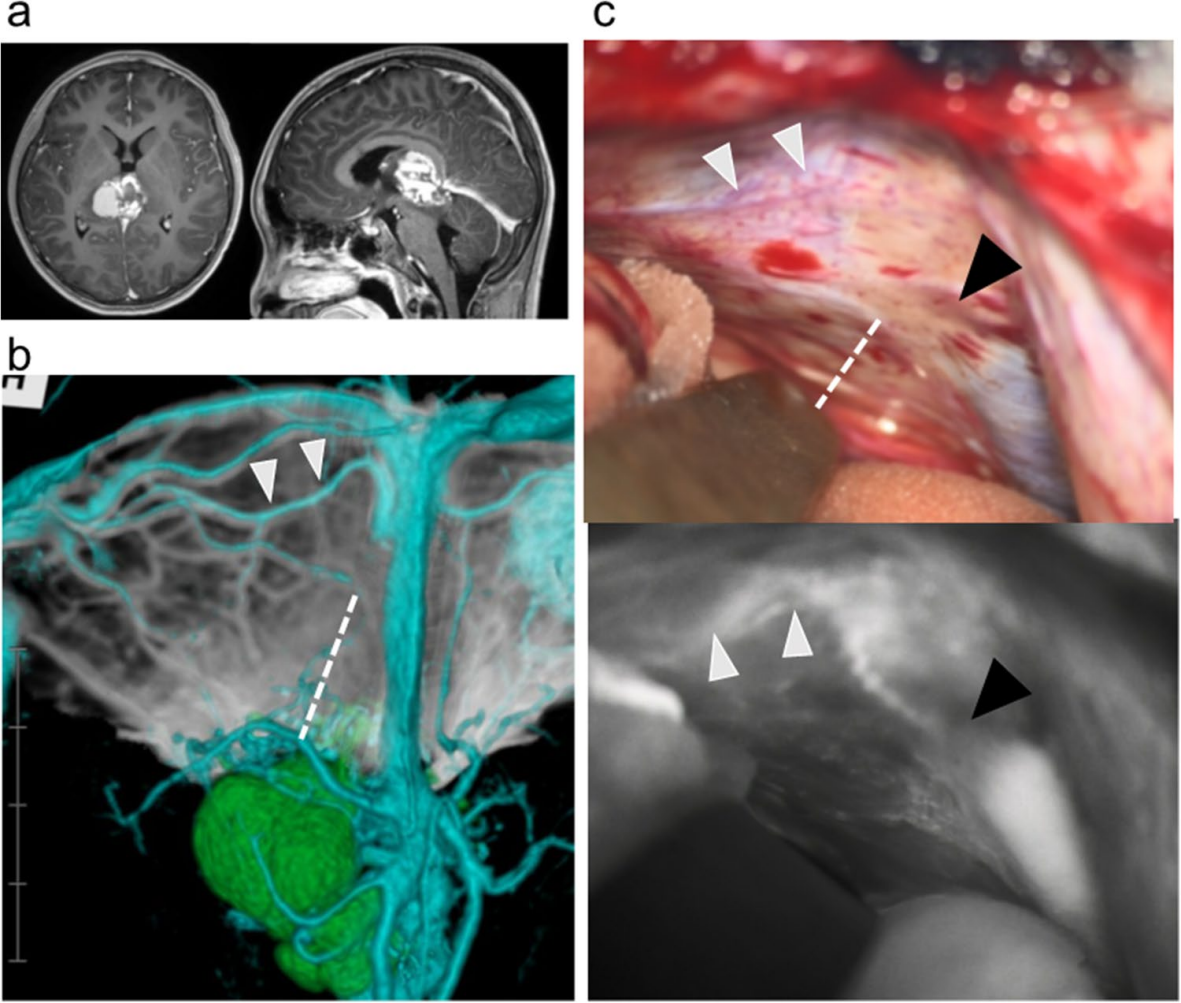
Case 7 with pineal immature teratoma. **a** Preoperative axial (left) and sagittal (right) gadolinium-enhanced T1-weighted MR images demonstrating the enhanced lesion in the pineal region. **b** Preoperative simulation of posterior views based on three-dimensional reconstruction with computed tomography venography and gadolinium-enhanced T1-weighted MR images demonstrating the anatomical relationships of the veins and sinuses (blue), tumor (green), and cerebellar tentorium (white). The dashed line indicates the expected tentorial incision. **c** Intraoperative findings of microscopy (upper panel) and indocyanine green video-angiography (lower panel) demonstrating that the tentorial sinus (white arrowhead), but not the proximal straight sinus (black arrowhead), was not visible on either modality. The dashed line in the upper panel indicates the actual tentorial incision

Additionally, simulation with the cerebellar tentorium provided information about the optimal tentorial incision, especially in cases with large tumor (Figs. 3 and 4 and Supplemental Fig. 1). The lesions were successfully exposed without complications caused by the selection of an inappropriate approach route in all cases.

### Estimation of preoperative simulation based on intraoperative findings

The preoperative simulation images identified all tentorial sinuses around the tentorial incision observed during surgery with the surgical microscope and ICG video-angiography in all eight sides examined (Figs. 3 and 4). The findings of preoperative simulation and intraoperative finding were consistent in all cases with regard to the presence or absence of tentorial sinus and its anatomical site (Table 1). However, the course of the straight sinus could not be recognized with the surgical microscope and ICG video-angiography in two sides because of the thickened dura mater (cases 1 and 7) (Fig. 4). Therefore, simulation imaging of the cerebellar tentorium is a useful complementary method for preoperative understanding of the anatomy of the tentorial and straight sinuses.

The relationships between the bridging veins and tentorial sinuses on the simulation images were classified as group I (Fig. 3) in four sides, group II (Supplementary Figs. 1 and 2) in nine sides, group III in three sides, groups I and II in one side, and group IV in one side (Fig. 1) (Table 1), and the patterns in the side of craniotomy were group I (Fig. 3) in two sides, group II in six sides (Fig. 2), and group III in one side. Intraoperative findings showed that the bridging vein from the occipital lobe was observed in both cases with group I (cases 2 and 7). In contrast, no bridging veins from the cerebellum were observed during OTA in the cases with group II because any potentially interfering bridging veins from the cerebellum were distant from the tentorial incision (cases 2, 4, 5, 6, and 8). Exceptionally, the bridging vein from the cerebellum was observed in the case with group II treated by suboccipital craniotomy (case 9) (Fig. 2). No bridging veins either from the cerebellum or occipital lobe were found in the case with group III. In total, the comparison of the simulation and intraoperative findings was consistent in all cases with regard to the presence or absence of bridging vein from occipital lobe (*n* = 8) and cerebellum (*n* = 1), and the site of bridging was accurately predicted by the simulation in all cases (Table 1).

These findings suggest that the simulation method provides accurate preoperative imaging of the relationships between the bridging vein and the tentorium, and no difficulty or complication was experienced during tentorial incision in the eight cases treated through the OTA.

## Discussion

The present study demonstrated that preoperative simulation imaging including the cerebellar tentorium based on Gd-3D-T1WI combined with routine vascular structure and tumor imaging obtained from CTA, CTV, and Gd-3D-T1WI could demonstrate the anatomical relationships between the tentorial sinus, bridging veins from the occipital lobe or cerebellum, deep veins, and tumor. This method can provide preoperative information about the optimum side and required extent of the tentorial incision, which was not provided by conventional CT venography without depiction of the cerebellar tentorium.

Previous reports have suggested that injury to the tentorial sinus itself does not lead to adverse consequences in most cases because of the collateral pathways, but that occlusion of the tentorial sinus can lead to major complications if the tentorial sinus acts as the main drainer due to occlusion of the main venous channels by the disease process [13, 14]. Additionally, we should consider the course of the basal veins and bridging veins draining into the tentorial sinus to avoid injury to the primitive tentorial sinus draining the basal vein [5, 13, 15] as shown in Fig. 1. Venous infarction in the occipital lobe and cerebellum, caused by injury of the bridging, is also one of the major complications of tumor resection in this region [3, 4, 9, 13, 14, 18–20]. Therefore, clear understanding the complex anatomical structures around the cerebellar tentorium is important to avoid this complication [4, 10, 13, 16, 17, 21].

Preoperative investigation has adopted angiography, CT, and MR imaging. Angiography can reveal real-time blood flow and is useful to detect any large feeding arteries of the tumor and the draining route. Recently, 3D CT and 3D MR imaging have become useful for preoperative assessment of the OTA through image processing to show the tumor localization, surrounding structures, and associated blood vessels [4, 11, 12]. 3D CTA demonstrated variations of the galenic system in 150 patients, and identified the variations requiring care at incision of the cerebellar tentorium as the basal vein draining into the persistent primitive tentorial sinus, and drainage of the internal occipital vein to the transverse sinus through the cerebellar tentorium [4]. 3D CT demonstrated the persistent primitive tentorial sinus in 10% of adult patients with unruptured aneurysm [5]. Furthermore, high-resolution 3D multifusion imaging combining with MR imaging, MR angiography, CT, and 3D rotational angiography was useful for preoperative simulation of hemangioblastoma, especially of the vascular structures [12].

Therefore, simulation of the vascular structures is known to have utility in the surgical planning of the OTA. In this study, we investigated the importance of inclusion of the cerebellar tentorium in the preoperative simulation of OTA. This technique had four advantages for the preoperative simulation of tentorial incision, comparing to that without the cerebellar tentorium. First, visualization of the cerebellar tentorium enabled identification of the bridging veins to the tentorial sinus, most commonly from the cerebellum, followed by the occipital lobe [13]. Preoperative evaluation of the presence and location of any cerebellar bridging veins behind the cerebellar tentorium is especially important because these veins are not visible before tentorial incision. Cerebellar bridging veins are most frequently located in the intermediate third and between the medial and intermediate borders of the cerebellar hemisphere on the left-right axis and intermediate third on the anteroposterior axis [14, 16]. Based on these anatomical characteristics, extension of the tentorial incision posteriorly has the risk of damaging the bridging vein from the cerebellum located under the cerebellar tentorium. To avoid this complication, preoperative simulation with the cerebellar tentorium will be useful in the surgical planning. Second, the basal vein sometimes drains into the tentorial sinus [4, 13] as in our case 4. The connections between the basal vein of rosenthal and tentorial sinus can be recognized by MR venography or CT angiography [4]. However, simulation with the cerebellar tentorium facilitated estimation of the risk of tentorial incision as shown in Fig. 1. In this way, the present simulation method allows individual determination of the feasibility of tentorial incision by showing the required range of incision and courses of the basal vein and tentorial sinus. Third, this simulation method provides clear information on the course of the straight sinus. ICG videoangiography is a useful method for detecting the parasagittal dural venous drainage and occipital sinus [22, 23]. However, no reports have demonstrated the diagnostic accuracy in the visualization of venous structures in the dura mater, and the straight sinus was invisible due to the thick dura mater in two of seven cases in this series. Therefore, preoperative assessment of straight sinus is necessary for safe tentorial incision at the moment, and previous reports have demonstrated the utility of CT and MR venography without the depiction of cerebellar tentorium [4, 24]. Compared to these conventional methods, simulation with the cerebellar tentorium provided the essential information for the optimal tentorial incision based on the anatomical relationship of the invisible straight sinus to the tentorial sinus, cerebellar tentorium, and tumor as shown in Fig. 4. This advantage will also potentially useful for the resection of extra-axial tumor with invasion to the straight sinus and confluence, but this study did not investigate this aspect. Fourth, the appropriate range of tentorial incision could be simulated based on the relationship of the cerebellar tentorium to the tumor and tentorial sinus, which was especially useful in cases of large tumor as shown in Figs. 3 and 4 and Supplemental Fig. 1). These advantages suggest that preoperative simulation including the cerebellar tentorium is useful for determining the optimum side and required extent of the tentorial incision in the OTA.

There are some limitations of this study. This simulation method provides accurate anatomical information about the bridging vein and tentorial sinus around cerebellar tentorium. However, these structures incorporate complex and individualized collateral pathways [16]. Therefore, this method has limits to predict venous infarction due to the preoperative workup based on CT and MR imaging [18]. Until imaging of the venous systems is improved, we agree with the strategy that focuses on preservation of the venous structure as much as possible based on the preoperative simulation and careful surgical strategies. Second, extraction of the cerebellar tentorium, vessels, and tumor was performed manually, and so, the simulation images took 40 min to reconstruct. Although our study demonstrates the concept and significance of simulation with the cerebellar tentorium in the planning of OTA, we have to develop the platform with excellent cost-effectiveness. To this end, development of rapid automatic segmentation and application to intraoperative navigation systems is indispensable. Recently, academic institutes and companies dealing with imaging software have rapidly developed the workstation [25]. Consequently, the present study provides a route to application of these technologies in the clinical practice.

In conclusion, preoperative simulation including the cerebellar tentorium is useful for determining the optimum and safe side and required extent of the tentorial incision necessary for tumor resection with the OTA.

### Supplementary information

ESM 1 (PNG 963 kb)

High resolution image (TIF 1046 kb)

ESM 2 (MP4 15426 kb)

ESM 3 (MP4 16716 kb)

## Data Availability

Not applicable.
