# Supplementary figures and images for: Simulation of the occipital transtentorial approach incorporating visualization of the cerebellar tentorium using three-dimensional computed tomography angiography and gadolinium-enhanced T1-weighted magnetic resonance imaging: technical note
Source: Neurosurg Rev. 2023 Sep 29;46(1):259. doi: 10.1007/s10143-023-02170-6 (PMC10542293; doi:10.1007/s10143-023-02170-6)

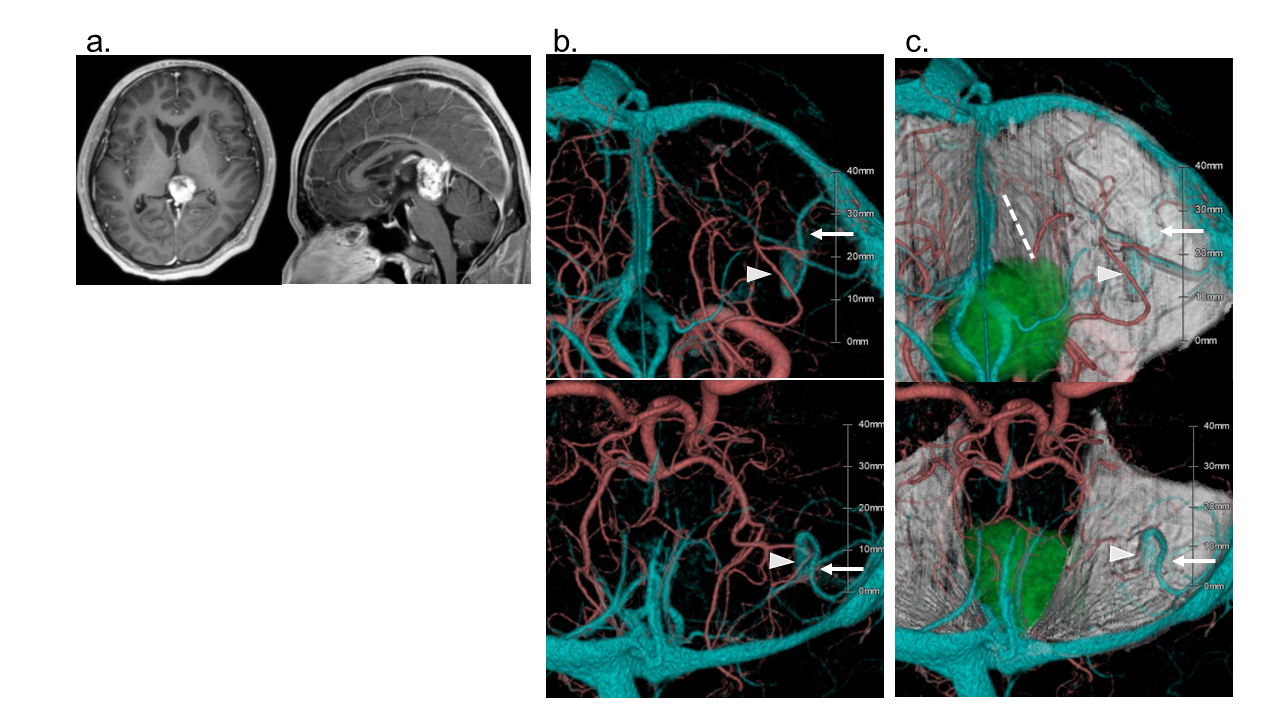

Supplement: Supplementary file 1 — (PNG 963 kb) [file 10143_2023_2170_Fig5_ESM.png]

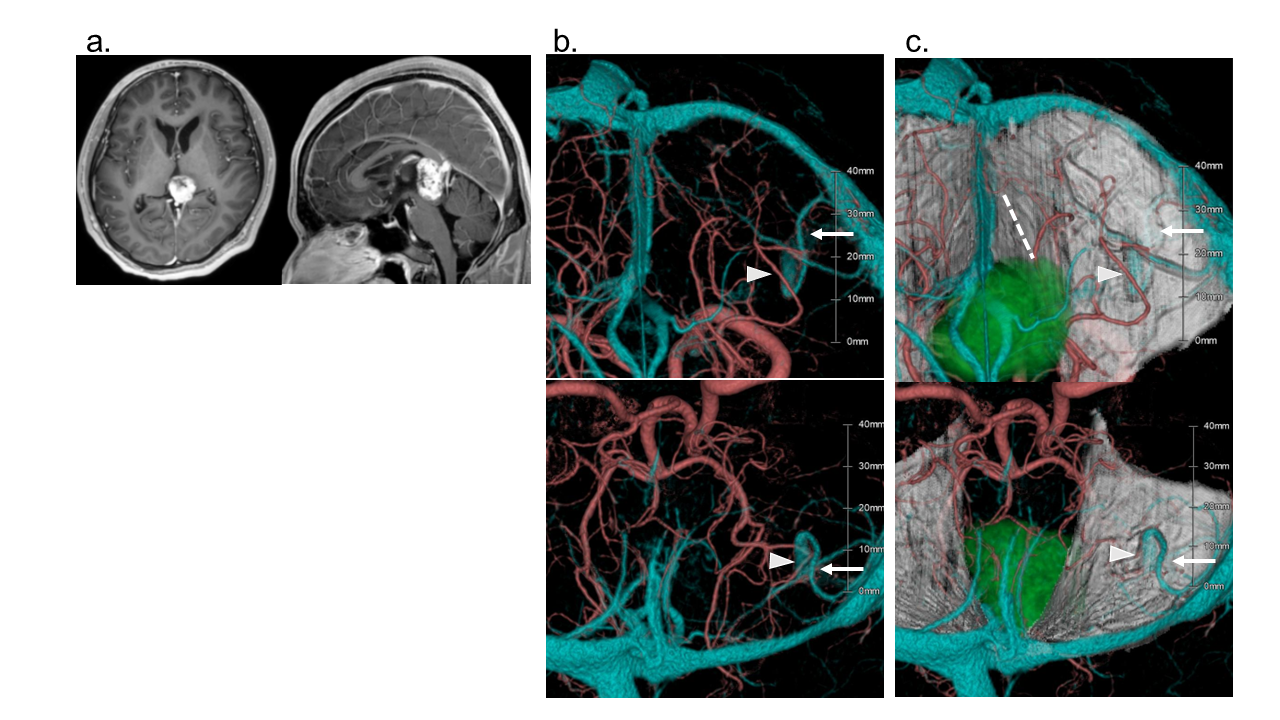

Supplement: Supplementary file 2 — High resolution image (TIF 1046 kb) [file 10143_2023_2170_MOESM1_ESM.tif]
